# Molecular characterization of invasive *Streptococcus pneumoniae* clinical isolates from a tertiary children’s hospital in eastern China

**DOI:** 10.1128/spectrum.00913-23

**Published:** 2023-09-27

**Authors:** Xu Huang, Hua Tan, Feng Lu, Genglin Guo, Mingxiao Han, Tongbo Cai, Haifang Zhang

**Affiliations:** 1 Department of Clinical Laboratory, Children’s Hospital of Nanjing Medical University, Nanjing, China; 2 School of Mechanical Engineering, Tongji University, Shanghai, China; 3 College of Veterinary Medicine, Nanjing Agricultural University, Nanjing, China; 4 Department of Clinical Laboratory, The Second Affiliated Hospital of Soochow University, Suzhou, China; 5 College of Computer and Information Engineering, Henan Normal University, Xinxiang, China; University of Maryland School of Medicine, Baltimore, Maryland, USA

**Keywords:** *S. pneumoniae*, IPD, WGS

## Abstract

*Streptococcus pneumoniae* is a common opportunistic pathogen that causes invasive pneumococcal disease (IPD), especially in children. This study aimed to determine the prevalence and molecular characteristics of *S. pneumoniae* isolated from children with IPD. A total of 78 *S*. *pneumoniae* isolates from aseptic body fluids of 70 IPD patients were collected at the Children’s Hospital of Nanjing Medical University (Jiangsu Province, China) during 2017–2021. Whole-genome sequencing technology was used to analyze the serotype, sequence type (ST), virulence, and antibiotic resistance of the 78 invasive *S. pneumoniae* clinical isolates. Our results showed that the pneumococcal infection rate declined after the COVID-19 outbreak in 2019. Serotypes 19F, 14, 6A, 23F, 19A, and 6B were the most common strains. The pneumococcal conjugate vaccine (PCV) 13 serotype coverage rate was 87.1%. All isolates were classified by multi-locus sequence typing (MLST) analysis into 27 different STs, including 3 novel STs (ST17941, ST17942, and ST17944) and 1 novel allele [recP (558)]. The most predominant ST was ST271, followed by ST320 and ST876. All isolates carried the following virulence genes: cbpG, lytB, lytC, pce (cbpE), pavA, slrA, plr (gapA), hysA, nanA, eno, piuA, psaA, cppA, iga, htrA (degP), tig (ropA), zmpB, and ply. All isolates were multidrug resistant and had high levels of resistance to macrolides, tetracyclines, and sulfonamides. Taken together, this study revealed extensive genetic diversity among *S. pneumoniae* isolates from a single Chinese hospital. Wearing masks, universal infant vaccination with PCV13, and the launch of recombinant protein vaccine development programs could reduce the burden of IPD in children.

**IMPORTANCE:**

Invasive pneumococcal disease (IPD) caused by *Streptococcus pneumoniae* in children remains a global burden and should be given more attention due to the fact that the pneumococcal vaccine is not fully covered globally. The molecular epidemiological characteristics of *S. pneumoniae* are not so clear, especially in these years of COVID-19. In this study, we collected *S. pneumoniae* isolates from the aseptic body fluid of children with IPD from 2017 to 2021 in a tertiary children’s hospital in China and revealed the extensive genetic diversity of these isolates. Most importantly, we first found that the rate of pneumococcal infection has declined since the COVID-19 outbreak in 2019, which means that wearing masks could reduce the transmission of *S. pneumoniae*. In addition, it was shown that universal infant vaccination with PCV13 seems essential for reducing the burden of IPD in children.

## INTRODUCTION

*Streptococcus pneumoniae* is a common Gram-positive coccus that colonizes the mucosal surface of the human upper respiratory tract. Asymptomatic colonization is especially high (27%–65%) in children (1). This etiologic agent may lead not only to respiratory tract infections but also to invasive pneumococcal disease (IPD), such as sepsis, meningitis, and pleurisy (2). IPDs are characterized by high morbidity and mortality rates worldwide and mainly affect children <5 years old, particularly in developing countries. The Global Burden of Diseases, Injuries, and Risk Factors Study 2017 estimated that approximately 380,900 children <5 years old die from pneumococcal disease. In the same year, *S. pneumoniae* was listed as 1 of the 12 priority pathogens by the World Health Organization (1).

There are at least 100 *S*. *pneumoniae* serotypes circulating worldwide. The biochemical structure of the capsular polysaccharides (CPS) serves as the basis for categorizing the *S. pneumoniae* serotypes (3). The CPS, as a virulence element affecting nearly every link of pneumococcal pathogenesis, are considered a prerequisite for IPD (4). Pneumococcal vaccines are designed and developed based on conjugated polysaccharides and are a remarkably effective means of preventing pneumococcal conjugate vaccine (PCV)-serotype pneumococcal disease. Indeed, timely tracing of the epidemiology of *S. pneumoniae* in children could contribute to the development of pneumococcal vaccine coverage.

*S. pneumoniae* virulence is attributed to CPS and multiple factors that mediate inflammatory responses via proteases serving dual roles (*S. pneumoniae* self-protein processors and host protein targets) (5). Virulence factors with unique biological functions mediate the adhesiveness and invasiveness of *S. pneumoniae*, as well as the pathogenesis underlying IPD (1). Virulence factors participate in inflammatory reactions at different infection sites, such as ClpP, BacB, and HtrA in the lung; CbpG, DacB, and ZmpC in the blood; and CbpA, ChoP, and PAFr at the blood-brain barrier (5, 6). Moreover, virulence factors have an impact on disease severity and the pathogenicity of the host (6). Therefore, monitoring the virulence profile is crucial and not only helps clinicians understand disease severity at an early stage but also facilitates the implementation of empirically targeted therapy.

The primary consideration in the clinical treatment of IPD is antimicrobial therapy. *S. pneumoniae* resistance to commonly used antibiotics, such as penicillins, macrolides, cephalosporins, and sulfonamides, has become a challenging problem, especially in Asian countries (7). Although large-scale pneumococcal vaccination has been introduced, *S. pneumoniae* drug resistance is still a very serious issue in countries where antibiotics are widely used, drug-resistant clones are widespread, and vaccines are less frequently administered. Therefore, it is essential to regulate antimicrobial resistance based on its regional status.

In the current study, whole-genome sequencing (WGS) was used to determine the prevalence and molecular characteristics of invasive *S. pneumoniae* strains isolated from patients admitted to the Children’s Hospital of Nanjing Medical University (Jiangsu Province, China) between 2017 and 2021. The aim of this study was to provide a vaccination and antibiotic therapy reference for the prevention and control of IPD in children.

## MATERIALS AND METHODS

### Bacterial isolates and clinical information

This study was conducted at the Children’s Hospital of Nanjing Medical University (Jiangsu Province, China) between 2017 and 2021. The Children’s Hospital of Nanjing Medical University is one of the largest pediatric hospitals in Jiangsu Province, with 1,742 beds and approximately 73,600 hospital admissions per year.

*S. pneumoniae* isolates were collected in this study when clinicians defined the isolates as infectious pathogens. Aseptic body fluids (e.g., cerebrospinal, blood, and pleural fluids) were collected from children infected with *S. pneumoniae* during hospitalization. Cultures were isolated on Columbia blood agar plates (Kemaja, Shanghai, China), which were incubated for 24–48 h in 5% carbon dioxide at 35°C. All isolates were identified as *S. pneumoniae* using a VITEK-2 compact system (bioMérieux, Marcy-l’Étoile, France) and optochin susceptibility testing.

The electronic case records, including pediatric patient demographics, specimen information, infection characteristics, antimicrobial treatment, and clinical outcome, were retrieved and reviewed.

The study was conducted in accordance with the Declaration of Helsinki and approved by the Clinical Trial Ethics Committee of the Children’s Hospital of Nanjing Medical University (No. 202301027-1).

### Genome sequencing, assembly, and annotation

Bacterial genomic DNA was extracted using the Omega Bio-Tek Bacterial DNA Kit (Omega Bio-Tek, Doraville, GA, USA). Whole genomes of *S. pneumoniae* were sequenced using a paired-end library with an average insert size of 350 bp (range, 150–600 kb) on an Illumina NovaSeq 6000 platform (Illumina Inc., San Diego, CA, USA). The genome data were assembled with Unicycler software (version 0.4.8; https://github.com/rrwick/Unicycler) for assembling genomes from a combination of short and long reads (8). All assembled genomes of *S. pneumoniae* were annotated using Prokka software (version 1.14.6; tseemann; https://github.com/tseemann/prokka).

### Serotyping

The *S. pneumoniae* serotypes were analyzed using Pneumococcal Capsular Typing (PneumoCaT, version 1.2.1; https://github.com/phe-bioinformatics/PneumoCaT), which used a two-step approach to assign capsular type to *S. pneumoniae* genomic data (9). In the first step, if the readset matched >90% to a single capsular locus sequence, the capsular type was reported; otherwise, the next step would commence. Serotypes within a serogroup/genogroup were distinguished by utilizing the capsular-type variant database.

### Multi-locus sequence typing

The sequence types (STs) of *S. pneumoniae* were analyzed utilizing multi-locus sequence typing (MLST) software (version 2.22.1, tseemann; https://github.com/tseemann/mlst). New alleles were amplified by polymerase chain reaction and sequenced using the Sanger method (10). Obtained sequences and strain-related information were submitted to the pneumococcal PubMLST database to automatically assign a new ST accession number.

The minimum spanning tree-like structures were drawn by PHYLOVIZ software (version 2.1; http://www.phyloviz.net) via the goeBURST algorithm (goeBURST distance) at SLV level 1 and the goeBURST Full MST algorithm (goeBURST distance) at LV level 6 (11).

### Phylogenetic analysis

The general feature format) files created by Prokka were analyzed using Roary (version 3.7.0; https://github.com/sanger-pathogens/Roary) to generate a core gene multiFASTA alignment (>99%) using multiple alignment using fast Fourier transform (12). Snp-sites (version 2.3.3; https://github.com/andrewjpage/snp-sites) was used to extract single nucleotide polymorphism (SNP) sites from the multiFASTA alignment (13). A maximum-likelihood tree was constructed from extracted SNPs utilizing RaxML software (version 8.2.12; https://github.com/stamatak/standard-RAxML), selecting the GTRGAMMA model. Eventually, the RaxML tree was visualized and annotated using an online tool (Interactive Tree Of Life, version 6; https://itol.embl.de).

### Gene analysis

*S. pneumoniae* virulence genes were screened by the virulence factor database [Virulence Factors of Bacterial Pathogens (mgc.ac.cn)], which features curating information relevant to bacterial pathogen virulence factors (14). Antimicrobial resistance genes following ERY resistance genes [erm (B), mef (A), and msr (D)] (15), TET resistance gene [tet (M)] (15), CHL resistance gene (cat-TC) (16), and extended-spectrum β-lactamase (blaTEM-116) (16) were screened using ABRicate software (version 1.0.1; tseemann; https://github.com/tseemann/abricate).

### Antibiotic susceptibility tests

The minimum inhibitory concentrations (MICs) of ceftriaxone (CRO), cefotaxime (CTX), meropenem (MEM), erythromycin (ERY), tetracycline (TET), chloramphenicol (CHL), moxifloxacin (MXF), levofloxacin (LVX), vancomycin (VAN), linezolid (LZD), and trimethoprim-sulfamethoxazole (SXT) were determined using the GP68 Test Kit by the VITEK-2 compact system (bioMérieux, Marcy-l’Étoile, France) according to the manufacturer’s instructions. The MIC for penicillin was detected using the *E*-test method (Antu, Zhenzhou, China). *S. pneumoniae ATCC49619* was used as quality control to ensure the accuracy of the results. Susceptibility interpretation followed the standards proposed by the 2021 Clinical and Laboratory Standards Institute (17).

### Statistical analysis

The Statistical Package for Social Sciences software (version 22.0; SPSS, Chicago, IL, USA) was used to analyze the statistical significance of the data. A chi-square test was used to determine whether the differences among the two groups were statistically significant. The Bonferroni method based on the chi-square test was used to determine whether the differences among multiple groups were statistically significant (16). The Bonferroni method compares a set of *n* tests in pairs and uses *P* < 0.05/*n* as the judgment criterion for correction (18).

## RESULTS

### Clinical characteristics

A total of 70 IPD cases were collected from the Children’s Hospital of Nanjing Medical University between 2017 and 2021. The strains isolated from eight children with double infections (bloodstream and cerebrospinal fluid) were enrolled in this study. Finally, we collected 78 *S*. *pneumoniae* isolates to conduct the study. The incidence of the cases in the past 5 years had a mountain-like profile: 2019 was the peak; the incidence of the cases had an upward trend before 2019; and the incidence of the cases had a downward trend after 2019. The demographic and clinical characteristics of the pediatric patients are shown in Table 1.

**TABLE 1:** Clinical characteristics of 70 pediatric patients

Characteristics	No. of patients	%
Total	70	100.0
Gender		
Male	39	55.7
Female	31	44.3
Age (months*)*		
<12	20	28.6
12–24	16	22.8
24–60	27	38.6
>60	7	10.0
Year		
2017	14	20.0
2018	14	20.0
2019	22	31.4
2020	10	14.3
2021	10	14.3
Season		
Spring	18	25.7
Summer	5	7.2
Autumn	21	30.0
Winter	26	37.1
Clinical department		
ICU	20	28.6
Emergency department	21	30.0
Internal medicine department	26	37.1
Surgical department	3	4.3
Sample type		
Total	78	100.0
Blood	52	66.7
Cerebrospinal fluid	24	30.8
Pleural fluid	2	2.5
Outcome		
Cure	54	77.1
Exacerbation and transfer	7	10.0
Dead/abandon^a^	9	12.9

^a^ Abandon means an outcome that pediatric patients rapidly deteriorate and parents have to give up treatment to request automatic discharge.

### Molecular serotyping and vaccine coverage rates

**FIG 1:**
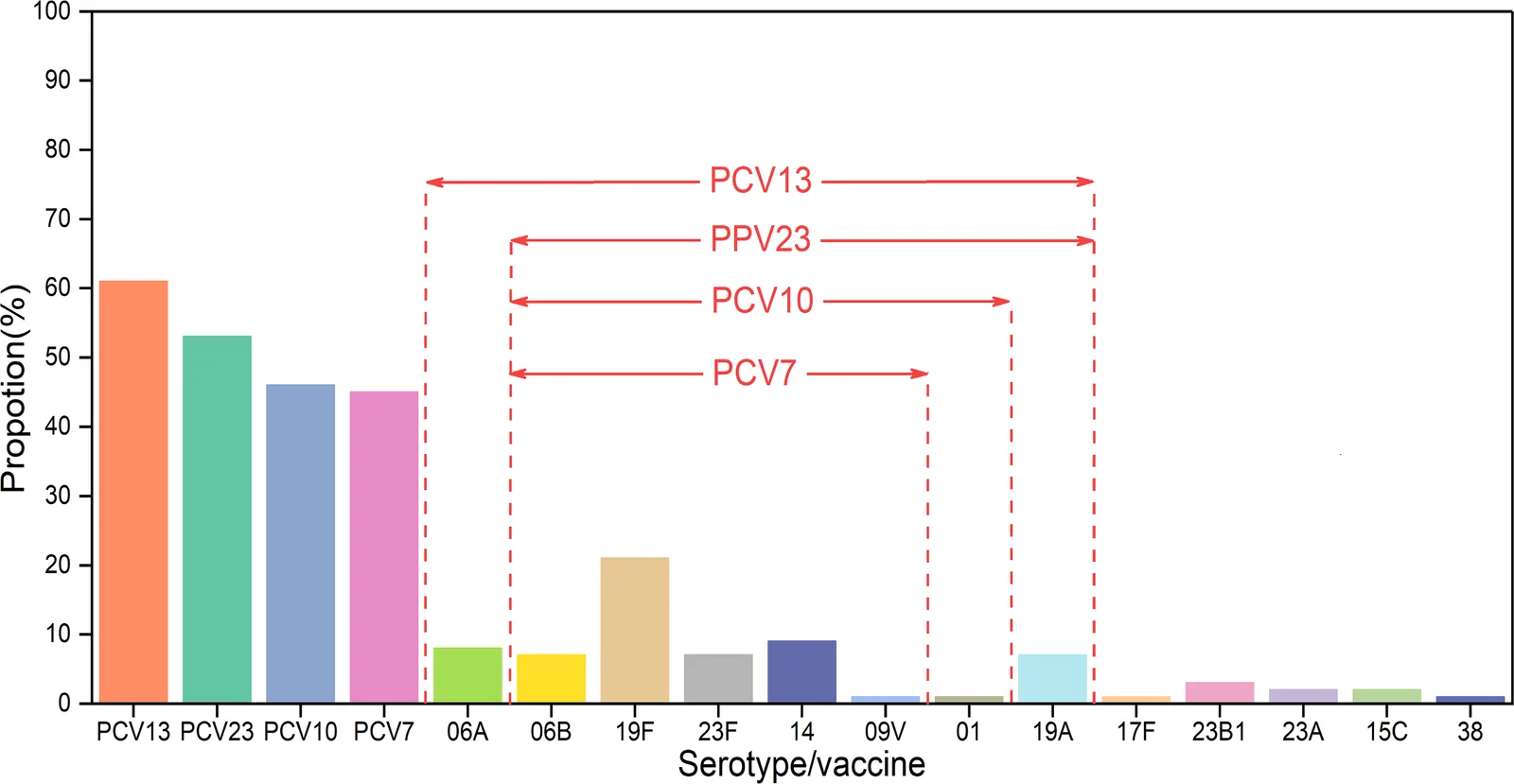
Serotype distribution and coverage of pneumococcal vaccines among *S. pneumoniae* isolates.

Considering that the isolates from the two specimen sources from each pediatric patient had the same serotype, we only included one isolate in the statistical analysis. All isolates were accurately classified into 13 different serotypes. The most predominant serotype was 19F (30.0%), followed by 14 (12.9%), 06A (11.4%), 23F (10.0%), 19A (10.0%), 06B (10.0%), 23B1 (4.3%), 23A (2.9%), 15C (2.9%), 09V (1.4%), 01 (1.4%), 17F (1.4%), and 38 (1.4%). Of all the isolates, the highest serotype coverage rate of the PCV13 vaccine was 87.1%, followed by PPV23 (75.7%), PCV10 (65.7%), and PPV7 (64.3%) (Fig. 1).

### MLST

Given that the isolates from the two specimen sources of each pediatric patient had the same ST, we only included one isolate in the statistical analysis. All isolates were classified by MLST analysis into 27 different STs, including 3 novel STs (ST17941, ST17942, and ST17944) and 1 novel allele [recP (558)]. The most predominant ST was ST271 [*n* = 18 (25.7%)], followed by ST320 [*n* = 7 (10.0%)], ST876 [*n* = 7 (10.0%)], ST90 [*n* = 5 (7.1%)], and ST902 [*n* = 5 (7.1%)]. Other STs collectively accounted for 40.0% of the isolates.

**FIG 2:**
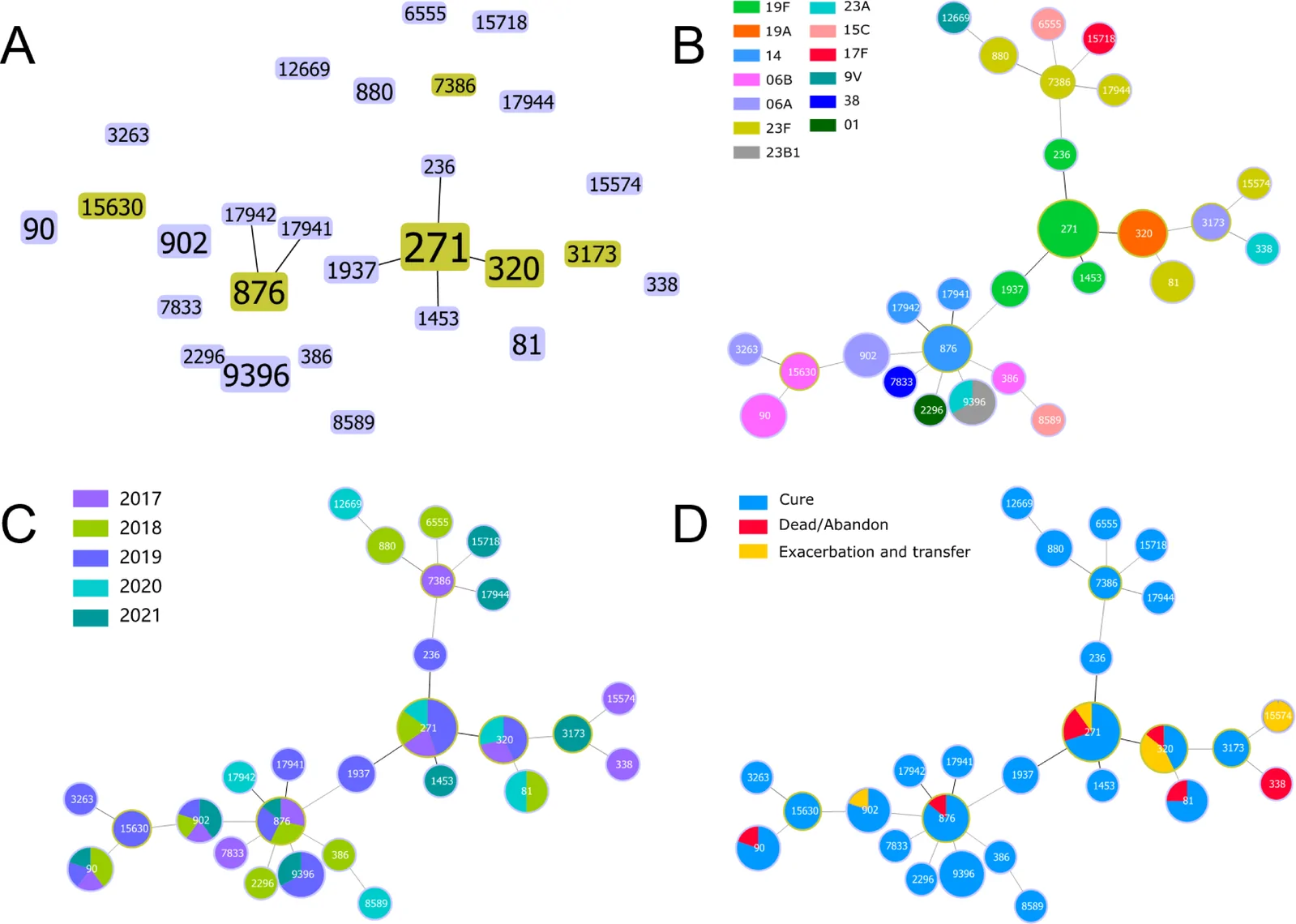
Minimum-spanning tree-like structure via the goeBURST full MST algorithm. (**A**) goeBURST distance at SLV level 1 showing that the 27 STs were divided into 2 CCs and 19 singletons. (**B**) goeBURST distance at LV level 6 showing the relationship between STs and serotypes. Each disk represents an ST, and each color represents a serotype. (**C**) The relationship between STs and years. (**D**) The relationship between STs and outcomes.

By comparing the strains with the pneumococcal molecular epidemiology network clones, two global clones with at least six of seven MLST alleles shared were identified in this study, including Spain23F-1 [*n* = 3 (4.2%)] and Taiwan19F-14 [*n* = 1 (1.4%)]. Two clonal complexes (CCs) and 19 singletons were obtained via the goeBURST algorithm (goeBURST distance) at SLV level 1 (Fig. 2A). CC271 (including ST271, ST1937, ST236, ST1453, and ST320) was the most prevalent CC, which accounted for 40.0% (*n* = 28/70) of the strains, followed by CC876 (including ST876, ST17941, and ST17942), which accounted for 12.9% (*n* = 9/70) of the strains.

The epidemiologic trends of MLST with serotype, year, and outcome were analyzed via the goeBURST Full MST algorithm (goeBURST distance) at LV level 6. Strains of the same serotype showed an aggregation trend in which ST271, ST320, ST876, ST90, and ST902 were associated with serotypes 19F, 19A, 14, 6B, and 06A, respectively (Fig. 2B). Moreover, the same ST-type strains mostly showed an annual aggregation distribution (Fig. 2C). Notably, most ST-type strains had a good outcome, but strains with a poor outcome tended to be concentrated in prevalent strains, such as ST271, ST320, and ST876 (Fig. 2D).

### Prevalence of virulence genes

**FIG 3:**
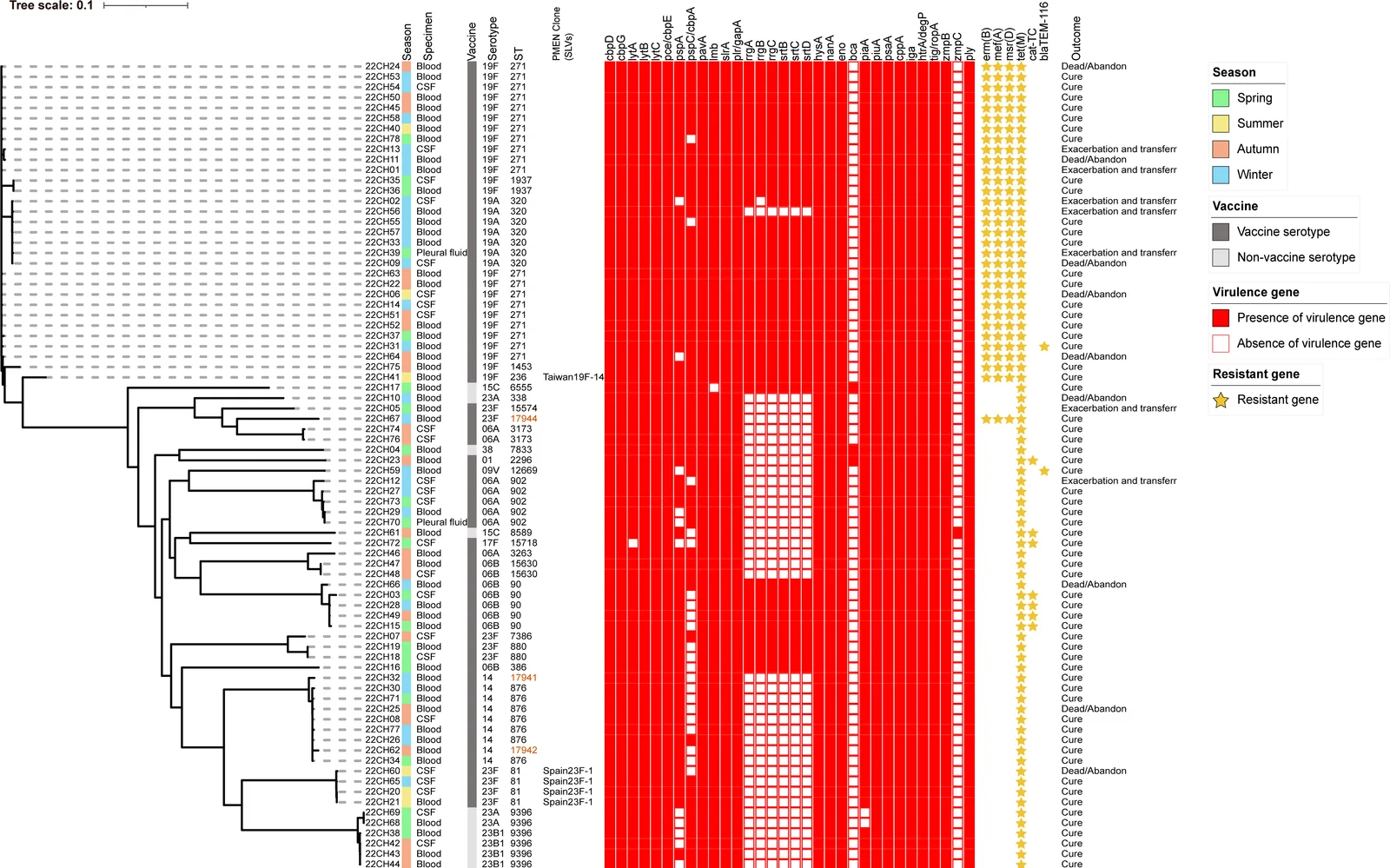
Maximum likelihood tree of 78 isolates. The RAxMl tree was constructed from SNPs using the GTRGAMMA method. The genetic characteristics of all isolates were annotated. ST, orange indicates the novel STs found in this study.

All 78 isolates carried cbpG, lytB, lytC, pce (cbpE), pavA, slrA, plr (gapA), hysA, nanA, eno, piuA, psaA, cppA, iga, htrA (degP), tig (ropA), zmpB, and ply. Most of the isolates carried cbpD (98.7%), lytA (98.7%), lmb (98.7%), piaA (97.4%), and pspA (85.9%). In addition, approximately one-half of the strains carried the virulence factor rlrA islets harboring related genes, including rrgA, rrgB, rrgC, strB, strC, and strD. The detailed information is shown in Table 2. The relationship between the isolates and virulence genes is shown in the annotation of the RAxML tree (Fig. 3). Seven of eight groups that were isolated from the two specimen sources of each pediatric patient harbored the same virulence genes; however, only one group showed a virulence gene profile difference (Fig. 3).

**TABLE 2:** Characteristics of virulence in 78 isolates^*a*^

VF class	Virulence factors	Related genes	Total	%	19F	%	23F	%	14	%	Others	%	*P* value	CC271	%	CC876	%	Others	%	*P* value
Adherence	Choline-binding proteins	cbpD	77	98.7	24	100.0	9	100.0	9	100.0	35	97.2	0.757	30	96.8	9	100.0	38	100.0	N.A.
		cbpG	78	100.0	24	100.0	9	100.0	9	100.0	36	100.0	N.A.	31	100.0	9	100.0	38	100.0	N.A.
		lytA	77	98.7	24	100.0	9	100.0	9	100.0	35	97.2	0.757	31	100.0	9	100.0	37	97.4	0.587
		lytB	78	100.0	24	100.0	9	100.0	9	100.0	36	100.0	N.A.	31	100.0	9	100.0	38	100.0	N.A.
		lytC	78	100.0	24	100.0	9	100.0	9	100.0	36	100.0	N.A.	31	100.0	9	100.0	38	100.0	N.A.
		pce/cbpE	78	100.0	24	100.0	9	100.0	9	100.0	36	100.0	N.A.	31	100.0	9	100.0	38	100.0	N.A.
		pspA	67	85.9	23	95.8	9	100.0	9	100.0	26	72.2	0.015^#^	29	93.5	9	100.0	29	76.3	0.054
		pspC/cbpA	57	73.1	**23**	**95.8**	**6^α^**	**66.7^α^**	**1^γ^**	**11.1^γ^**	**27^α^**	**75.0^α^**	0.000*	**29**	**93.5**	**1^γ^**	**11.1^γ^**	**27^β^**	**71.1^β^**	0.000*
	Fibronectin-binding proteins	pavA	78	100.0	24	100.0	9	100.0	9	100.0	36	100.0	N.A.	31	100.0	9	100.0	38	100.0	N.A.
	Laminin-binding protein	lmb	77	98.7	24	100.0	9	100.0	9	100.0	35	97.2	0.757	31	100.0	9	100.0	37	97.4	0.587
	Streptococcal lipoprotein rotamase A	slrA	78	100.0	24	100.0	9	100.0	9	100.0	36	100.0	N.A.	31	100.0	9	100.0	38	100.0	N.A.
	Streptococcal plasmin receptor/GAPDH	plr/gapA	78	100.0	24	100.0	9	100.0	9	100.0	36	100.0	N.A.	31	100.0	9	100.0	38	100.0	N.A.
	rlrA islet	rrgA	40	51.3	**24**	**100.0**	**3^γ^**	**33.3^γ^**	**0^γ^**	**0.0^γ^**	**13^γ^**	**36.1^γ^**	0.000*	**30**	**96.8**	**0^γ^**	**0.0^γ^**	**10^γ^**	**26.3^γ^**	0.000*
		rrgB	39	50.0	**24**	**100.0**	**3^γ^**	**33.3^γ^**	**0^γ^**	**0.0^γ^**	**12^γ^**	**33.3^γ^**	0.000*	**29**	**93.5**	**0^γ^**	**0.0^γ^**	**10^γ^**	**26.3^γ^**	0.000*
		rrgC	40	51.3	**24**	**100.0**	**3^γ^**	**33.3^γ^**	**0^γ^**	**0.0^γ^**	**13^γ^**	**36.1^γ^**	0.000*	**30**	**96.8**	**0^γ^**	**0.0^γ^**	**10^γ^**	**26.3^γ^**	0.000*
		srtB	40	51.3	**24**	**100.0**	**3^γ^**	**33.3^γ^**	**0^γ^**	**0.0^γ^**	**13^γ^**	**36.1^γ^**	0.000*	**30**	**96.8**	**0^γ^**	**0.0^γ^**	**10^γ^**	**26.3^γ^**	0.000*
		srtC	40	51.3	**24**	**100.0**	**3^γ^**	**33.3^γ^**	**0^γ^**	**0.0^γ^**	**13^γ^**	**36.1^γ^**	0.000*	**30**	**96.8**	**0^γ^**	**0.0^γ^**	**10^γ^**	**26.3^γ^**	0.000*
		srtD	40	51.3	**24**	**100.0**	**3^γ^**	**33.3^γ^**	**0^γ^**	**0.0^γ^**	**13^γ^**	**36.1^γ^**	0.000*	**30**	**96.8**	**0^γ^**	**0.0^γ^**	**10^γ^**	**26.3^γ^**	0.000*
Enzyme	Hyaluronidase	hysA	78	100.0	24	100.0	9	100.0	9	100.0	36	100.0	N.A.	31	100.0	9	100.0	38	100.0	N.A.
	Neuraminidase A	nanA	78	100.0	24	100.0	9	100.0	9	100.0	36	100.0	N.A.	31	100.0	9	100.0	38	100.0	N.A.
	Streptococcal enolase	eno	78	100.0	24	100.0	9	100.0	9	100.0	36	100.0	N.A.	31	100.0	9	100.0	38	100.0	N.A.
Immunore active antigen	Alpha C protein	bca	3	3.8	0	0.0	0	0.0	0	0.0	3	8.3	0.303	0	0.0	0	0.0	3	7.9	0.194
Iron uptake	Pneumococcal iron acquisition	piaA	76	97.4	24	100.0	9	100.0	9	100.0	34	94.4	0.495	31	100.0	9	100.0	36	94.7	0.339
	Pneumococcal iron uptake	piuA	78	100.0	24	100.0	9	100.0	9	100.0	36	100.0	N.A.	31	100.0	9	100.0	38	100.0	N.A.
Manganese uptake	Pneumococcal surface antigen A/metal-binding protein SloC	psaA	78	100.0	24	100.0	9	100.0	9	100.0	36	100.0	N.A.	31	100.0	9	100.0	38	100.0	N.A.
Protease	C3-degrading protease	cppA	78	100.0	24	100.0	9	100.0	9	100.0	36	100.0	N.A.	31	100.0	9	100.0	38	100.0	N.A.
	IgA1 protease	iga	78	100.0	24	100.0	9	100.0	9	100.0	36	100.0	N.A.	31	100.0	9	100.0	38	100.0	N.A.
	Serine protease	htrA/degP	78	100.0	24	100.0	9	100.0	9	100.0	36	100.0	N.A.	31	100.0	9	100.0	38	100.0	N.A.
	Trigger factor	tig/ropA	78	100.0	24	100.0	9	100.0	9	100.0	36	100.0	N.A.	31	100.0	9	100.0	38	100.0	N.A.
	Zinc metalloproteinase	zmpB	78	100.0	24	100.0	9	100.0	9	100.0	36	100.0	N.A.	31	100.0	9	100.0	38	100.0	N.A.
		zmpC	1	1.3	0	0.0	0	0.0	0	0.0	1	2.8	0.757	0	0.0	0	0.0	1	2.6	0.587
Toxin	Pneumolysin	ply	78	100.0	24	100.0	9	100.0	9	100.0	36	100.0	N.A.	31	100.0	9	100.0	38	100.0	N.A.

^*a*^ The characteristics of virulence genes in 78 strains with different serotypes and CCs. The Bonferroni method based on the chi-square test was used to test whether the differences among multiple groups were statistically significant. According to the Bonferroni method, when *P* < 0.0166, the differences among three groups were statistically significant; when *P* < 0.0125, the differences among four groups were statistically significant. “Bold” means that the difference is statistically significant, and “N.A.” means no difference among multiple groups. “*” indicates *P* < 0.05. “#” indicates the situation where *P* < 0.05 but does not match the chi-square test with the Bonferroni method. The symbols (α, β,γ) represent the groups with significant differences. Compare multiple sets of data in pairs based on the first group in the Bonferroni method: *P* < 0.05/*n*, “γ” means significant differences; *P* = 0.05/*n*, “β” means unable to determine; *P* > 0.05/*n*, “α” means no significant differences.

There was a statistically significant association between some serotypes and virulence genes, as shown in Table 2. Among adherence factors, the rlrA islet, as one of the virulence factors, had a considerable connection with serotype 19F. Related genes, including rrgA, rrgB, rrgC, strB, strC, and strD, were prevalent in serotype 19F but rarely detected in serotypes 23F, 14, and the remaining 10 detected serotypes (100.0% vs 33.3% vs 0.0% vs 36.1%; *P* = 0.000). In addition, the carriage rate of pspC/cbpA was highest in serotype 19F but lowest in serotypes 23F, 14, and the remaining 10 detected serotypes (95.8% vs 66.7% vs 11.1% vs 75.0%; *P* = 0.000).

Among the strains of different CCs, the related gene-carrying rates varied, as shown in Table 2. For adherence factors in particular, the pspC/cbpA carriage rate was highest in CC271 and lowest in CC876 (93.5% vs 11.1%; *P* = 0.000).

### Relationship between antibiotic susceptibility and molecular characteristics

The antibiotic resistance phenotypes and genes of 78 *S*. *pneumoniae* isolates are displayed in Table 3. As shown in Table 3, all of the strains isolated from patients with meningitis were resistant to PEN, while the strains isolated from non-meningitis patients were susceptible to PEN. The CRO and CTX resistance rates of the strains isolated from meningitis patients were significantly higher than the strains isolated from non-meningitis patients (16.7% vs 14.8% and 41.7% vs 18.5%, respectively; *P* = 0.000). Most isolates showed high resistance to ERY (100.0%) and TET (93.6%), and a considerable number of isolates were resistant to SXT (69.2%). None of the strains were resistant to LZD or VAN. All of the isolates exhibiting resistance to at least one antimicrobial drug in three or more antimicrobial categories were defined as having multidrug resistance (MDR). Notably, there were differences in the drug sensitivity results of the strains isolated from different specimen sources for each pediatric patient.

**TABLE 3:** Antibiotic resistance and resistance-related genes in 78 isolates^*a*^

		Total		19F		23F		14		Others		*P* value	CC271		CC876		Others		*P* value
		*R*	%	*R*	%	*R*	%	*R*	%	*R*	%		*R*	%	*R*	%	*R*	%	
PEN	Meningitis (*n* = 24)^a^	24	100.0	6	100.0	5	100.0	1	100.0	12	100.0	N.A.	8	100.0	1	100.0	15	100.0	N.A.
	Non-meningitis (*n* = 54)^b^	0	0.0	0	0.0	0	0.0	0	0.0	0	0.0	N.A.	0	0.0	0	0.0	0	0.0	N.A.
CRO	Meningitis (*n* = 24)^a^	4	16.7	3	50.0	1	20.0	0	0.0	0	0.0	0.059	**4**	**50.0**	**0** *^α^*	**0.0** *^α^*	**1** *^β^*	**6.7** *^β^*	**0.045^#^**
	Non-meningitis (*n* = 54)^b^	9	14.8	**9**	**50.0**	**0** *^α^*	**0.0** *^α^*	**0** *^α^*	**0.0** *^α^*	**0** *^γ^*	**0.0** *^γ^*	0.00*	**8**	**34.7**	**0** *^α^*	**0.0** *^α^*	**0** *^γ^*	**0** *^γ^*	0.002*
CTX	Meningitis (*n* = 24)^a^	10	41.7	**5**	**83.3**	**2** *^α^*	**40.0** *^α^*	**1** *^α^*	**100.0** *^α^*	**2** *^γ^*	**16.7** *^γ^*	**0.032** ^#^	5	62.5	1	100.0	4	26.7	0.121
	Non-meningitis (*n* = 54)^b^	11	18.5	**11**	**61.1**	**0** *^α^*	**0.0** *^α^*	**0** *^γ^*	**0.0** *^γ^*	**0** *^γ^*	**0.0** *^γ^*	0.00*	**11**	**47.8**	**0** *^γ^*	**0.0** *^γ^*	**0** *^γ^*	**0.0** *^γ^*	0.00*
MEM		10	12.8	6	25.0	0*^α^*	0.0*^α^*	2*^α^*	22.2*^α^*	2*^α^*	5.6*^α^*	0.074	6	19.4	2	22.2	2	5.3	0.147
ERY		78	100.0	24	100.0	9	100.0	9	100.0	36	100.0	N.A.	31	100.0	9	100.0	38	100.0	N.A.
TET		73	93.6	**23**	**95.8**	**9** *^α^*	**100.0** *^α^*	**5** *^γ^*	**55.6** *^γ^*	**36** *^α^*	**100.0** *^α^*	0.00*	**30**	**96.8**	**5** *^γ^*	**55.6** *^γ^*	**38** *^α^*	**100.0** *^α^*	0.00*
CHL		7	9.0	**0**	**0.0**	**0**	**0.0**	**0**	**0.0**	**7** *^α^*	**19.4** *^α^*	**0.03** ^#^	**0**	**0.0**	**0**	**0.0**	**7** *^γ^*	**18.4** *^γ^*	**0.017^#^**
MXF		25	32.1	6	25.0	5	55.6	2	22.2	12	33.3	0.354	8	25.8	2	22.2	15	39.5	0.384
LVX		25	32.1	6	25.0	5	55.6	21	22.2	12	33.3	0.354	8	25.8	2	22.2	15	39.5	0.384
VAN		0	0.0	0	0.0	0	0.0	0	0.0	0	0.0	N.A.	0	0.0	0	0.0	0	0.0	N.A.
LZD		0	0.0	0	0.0	0	0.0	0	0.0	0	0.0	N.A.	0	0.0	0	0.0	0	0.0	N.A.
SXT		54	69.2	**23**	**95.8**	**5** *^γ^*	**55.6** *^γ^*	**0** *^γ^*	**0.0** *^γ^*	**26** *^α^*	**72.2** *^α^*	0.00*	**30**	**96.8**	**0** *^γ^*	**0.0** *^γ^*	**24** *^γ^*	**63.2** *^γ^*	0.00*
erm (B)		78	100.0	24	100.0	9	100.0	9	100.0	36	100.0	N.A.	31	100.0	9	100.0	38	100.0	N.A.
mef (A)		32	41.0	**24**	**100.0**	**1** *^γ^*	**11.1** *^γ^*	**0** *^γ^*	**0.0** *^γ^*	**7** *^γ^*	**19.4** *^γ^*	0.00*	**31**	**100.0**	**0** *^γ^*	**0.0** *^γ^*	**1** *^γ^*	**2.6** *^γ^*	0.00*
msr (D)		32	41.0	**24**	**100.0**	**1** *^γ^*	**11.1** *^γ^*	**0** *^γ^*	**0.0** *^γ^*	**7** *^γ^*	**19.4** *^γ^*	0.00*	**31**	**100.0**	**0** *^γ^*	**0.0** *^γ^*	**1** *^γ^*	**2.6** *^γ^*	0.00*
tet (M)		78	100.0	24	100.0	9	100.0	9	100.0	36	100.0	N.A.	31	100.0	9	100.0	38	100.0	N.A.
cat-TC		7	9.0	**0**	**0.0**	**0**	**0.0**	**0**	**0.0**	**7** *^α^*	**19.4** *^α^*	**0.03^#^**	**0**	**0.0**	**0**	**0.0**	**7** *^γ^*	**18.4** *^γ^*	**0.017^#^**
blaTEM-116		2	2.6	1	4.2	0	0.0	0	0.0	1	2.8	0.867	1	3.2	0	0.0	1	2.6	0.864

^*a*^ Antibiotic resistance and resistance genes in 78 strains with different serotypes and CCs. The chi-square test was used to test whether the differences between two groups were statistically significant. The Bonferroni method based on the chi-square test was used to test whether the differences among multiple groups were statistically significant. According to the Bonferroni method, when *P* < 0.0166, the differences among three groups were statistically significant; when *P* < 0.0125, the differences among four groups were statistically significant. “Bold” means the difference is statistically significant, and “N.A.” means no difference among multiple groups. “*” indicates *P* < 0.05/*n*. “^#^” indicates the situation where 0.05/*n* < *P* < 0.05, which means that the *P* value matches *P* < 0.05 but does not match the chi-square test with the Bonferroni method. The symbols (α, β,γ) represent the groups with significant differences. Compare multiple sets of data in pairs based on the first group in the Bonferroni method: *P* < 0.05/*n*, “*γ*” means significant differences; *P* = 0.05/*n*, “*β*” means unable to determine; *P* > 0.05/*n*, “*α*” means no significant differences. a, meningitis breakpoint; b, non-meningitis breakpoint. PEN, penicillin; VAN, vancomycin. Resistance genes including erm (B), mef (A), and msr (D), TET resistance gene tet (M), CHL resistance gene cat-TC, and expended-spectrum β-lactamase blaTEM-116 were antibiotic resistance-related genes.

The SXT resistance rates of the serotype 19F strains were significantly higher than the other serotype strains, accounting for 95.8%. The antibiotic resistance phenotype varied by two CCs. For example, the TET and SXT resistance rates of the CC271 strains were markedly higher than the CC876 strains (96.8% vs 55.6%; *P* = 0.001 and 96.8% vs 0%; *P* = 0.000).

All of the isolates carried the ERY resistance gene erm (B) and the TET resistance gene tet (M). Both ERY resistance genes, mef (A) and msr (D), had the same carrier rate (41%). Some isolates carried the CHL resistance gene, cat-TC [9% (*n* = 7)], and the expended-spectrum β-lactamase, blaTEM-116 [2.6% (*n* = 2)]. In addition, all 19F serotypes and CC271 isolates simultaneously harbored the genes, including erm (B), mef (A), msr (D), and tet (M).

## DISCUSSION

Pediatric IPD remains a global burden, although the pneumococcal disease mortality rate in children has declined substantially in the pneumococcal vaccine era (19). Pediatric IPD should be given more attention because the pneumococcal vaccine is not administered universally in China. Indeed, the Children’s Hospital of Nanjing Medical University is the only children’s medical center in Jiangsu Province that receives nearly all pediatric patients throughout the province. Thus, this study accurately reflects the epidemiology of IPD in children within the entire Jiangsu Province. In the current study, the pneumococcal infection rate declined after the COVID-19 outbreak in 2019, which indicates that wearing masks prevents *S. pneumoniae* droplet transmission and reduces the infection rate. In fact, a recent survey showed that *S. pneumoniae* was the most common co-infected bacteria among COVID-19 patients in Jiangsu Province in 2020 (20). Therefore, it is essential to conduct an epidemiologic investigation of IPD in children.

The distribution of serotypes varies with geographic region, age, infection type, and time. In this study, the most prevalent distribution of serotypes among invasive *S. pneumoniae*-infected children in Nanjing was 19F, 14, 6A, 23F, 19A, and 6B, which were similar to other geographic regions of China, such as southwest China (19F, 19A, 6B, 6A, and 14), East China (19F, 6A/B, 23F, 14, and 15A), Shanghai (19F, 19A, 6A, 14, and 6B), and Beijing (19F, 19A, 23F, 14, and 6A) (15, 16, 21, 22). Conversely, there were differences in the serotype distribution in international regions, such as Africa (28F, 6A, 11A, 3, and 7C) and Egypt (1, 6ABC, 19F, 5, and 18ABC) (3, 23, 24). The serotype distribution discrepancy in different countries may be due to the genetic variation of *S. pneumoniae* or vaccination coverage. In addition, the serotype distribution between adults and children was significantly different. The most predominant serotypes among adults with pneumonia were 35B, 3, 11A/D, and 23A in the United States; 3, 8, 15A, 12F, and 11A in the UK; and 12F, 3, 23A, 19A, and 10A in Japan (25 – 27). It is, therefore, suggested that the prevalent serotypes not covered by a vaccine should be considered in future recombinant protein vaccine research and development plans. The PCV13 serotype coverage in invasive isolates was not only higher than a multi-center study conducted in China (73.3%) but also higher than other developed countries (Spain, 41%; Japan, 28.2%) (10, 27, 28). Although pneumococcal-related vaccines have been introduced in China for a long time, pneumococcal vaccination is still a non-program immunization vaccine, according to the principle of informed and voluntary use. Therefore, strengthening the awareness of vaccine protection in China, especially in Jiangsu, would help reduce the *S. pneumoniae* infection rate.

MLST analysis showed that ST271, ST320, and ST876 were the most predominant STs in our study, which were similar to the STs in other regions of China (16). The current study identified three novel STs and one novel allele, indicating that there is variation in the genetic evolution of *S. pneumoniae*. In the phylogenetic tree analysis, there were only two clonal complexes (CC271 and CC876) relatively less than in other regions of China (10). This phenomenon with regional features may be attributed to the individual hospital analysis rather than a multicenter study. Moreover, ST271, ST320, and ST876 were shown to be correlated with serotypes 19F, 19A, and 14, which are in agreement with the STs previously reported (16). Phylogenetic tree analysis also revealed that most ST strains manifested a good outcome; only a few ST strains, including ST320, ST271, and ST876, may lead to a poor outcome.

*S. pneumoniae* can carry dozens of virulence factors, which can be divided into the following categories according to functions: (i) the capsular polysaccharide; (ii) adherence; (iii) enzyme; (iv) immunoreactive antigen; (v) iron uptake; (vi) manganese uptake; (vii) protease; and (viii) toxin. These virulence factors mediate the entire pathogenesis process, including colonization, establishment of disease, and immune subversion. All isolates in our study carried cbpG, lytB, lytC, pce (cbpE), pavA, slrA, plr (gapA), hysA, nanA, eno, piuA, psaA, cppA, iga, htrA (degP), tig (ropA), zmpB, and ply. Apparently, these virulence-related genes are involved in the entire basic pathogenic process. First, virulence-related genes, including cbpG, lytB, lytC, pce (cbpE), pavA, slrA, and plr (gapA), contribute to adherence and colonization (5, 6). The next step is the accumulation of *S. pneumoniae* in the human circulatory system by various means, such as adhering to the airway epithelium (nanA), inhibiting recognition by C-reactive protein, neutralizing the antimicrobial peptide lactoferricin (psaA), and exerting cytotoxic effects (ply) (5, 29, 30). Most importantly, these conserved virulence genes exist in each strain, which may make a contribution to the development of virulence protein-based vaccines. Indeed, all serotype 19F strains in our study harbor the rlrA pathogenicity islet-related genes, which indicated that there may be a strong correlation.

The status of *S. pneumoniae* resistance is still serious in China, which reflects the widespread use of antibiotics, the large spread of drug-resistant clones, and low vaccination coverage (7). Beta-lactam antibiotics are widely recognized as an important choice to treat invasive *S. pneumoniae* infection (31, 32). Our study revealed the opposite result that the *S. pneumoniae* resistance rate to penicillin was 0% in non-meningitis patients but 100% in meningitis patients. The results suggest that penicillin could be used as a first-line antibiotic for children with invasive *S. pneumoniae* non-meningitis infection, rather than a meningitis infection. In agreement with previous studies, our study showed that serotype 19F strains had an apparent higher resistance rate to β-lactam antibiotics than others, which was associated with the spread of the resistant clone CC271. Apart from this, all isolates harboring erm (B) and tet (M) genes showed extremely high resistance to ERY and TET. Clearly, all isolates were sensitive to both VAN and LZD, which suggested that VAN and LZD could be alternatives for empiric treatment.

The highlight of this study was the utilization of WGS technology to determine the prevalence and molecular characteristics of invasive *S. pneumoniae* strains from children in Jiangsu Province, China. This study not only filled the gaps in the pediatric pneumococcal database of Jiangsu Province but also provided a reference for clinicians in this region to treat IPD. However, this study only included one children’s hospital, resulting in a small sample size with which we cannot compare annualized differences. Although the isolates from two specimen sources of each pediatric patient were confirmed to be the same serotype and ST, there were differences in the virulence gene profile and drug sensitivity results, the reasons for which need further research.

In summary, this study provided a systemic insight into the epidemiologic investigation of *S. pneumoniae* strains causing IPD in children. The rate of pneumococcal infection in our study has declined since the COVID-19 outbreak in 2019, so wearing masks could reduce the transmission of *S. pneumoniae*. Given the high PCV13 coverage rate and MDR full coverage, universal infant vaccination with PCV13 seems essential in this region. Finally, all isolates carried virulence genes, which could inspire recombinant protein vaccine development programs in the future.

## Data Availability

All of the genomes sequenced in this study were submitted to GenBank under accession number PRJNA891432.

## References

[1] Weiser JN , Ferreira DM , Paton JC . 2018. Streptococcus pneumoniae: transmission, colonization and invasion. Nat Rev Microbiol 16:355–367. doi:10.1038/s41579-018-0001-8 29599457 PMC5949087

[2] Bogaert D , De Groot R , Hermans PWM . 2004. Streptococcus pneumoniae colonisation: the key to pneumococcal disease. Lancet Infect Dis 4:144–154. doi:10.1016/S1473-3099(04)00938-7 14998500

[3] El-Kholy A , Badawy M , Gad M , Soliman M . 2020. Serotypes and antimicrobial susceptibility of nasopharyngeal isolates of Streptococcus pneumoniae from children less than 5 years old in Egypt. Infect Drug Resist 13:3669–3677. doi:10.2147/IDR.S250315 33116686 PMC7586055

[4] Weinberger DM , Trzciński K , Lu Y-J , Bogaert D , Brandes A , Galagan J , Anderson PW , Malley R , Lipsitch M . 2009. Pneumococcal capsular polysaccharide structure predicts serotype prevalence. PLoS Pathog 5:e1000476. doi:10.1371/journal.ppat.1000476 19521509 PMC2689349

[5] Marquart ME . 2021. Pathogenicity and virulence of Streptococcus pneumoniae: cutting to the chase on proteases. Virulence 12:766–787. doi:10.1080/21505594.2021.1889812 33660565 PMC7939560

[6] Loughran AJ , Orihuela CJ , Tuomanen EI . 2019. Streptococcus pneumoniae: invasion and inflammation. Microbiol Spectr 7. doi:10.1128/microbiolspec.GPP3-0004-2018 PMC6422050 30873934

[7] Page AJ , Taylor B , Delaney AJ , Soares J , Seemann T , Keane JA , Harris SR . 2016. SNP-sites: rapid efficient extraction of SNPs from multi-FASTA alignments. Microb Genom 2:e000056. doi:10.1099/mgen.0.000056 28348851 PMC5320690

[8] Wick RR , Judd LM , Gorrie CL , Holt KE . 2017. Unicycler: resolving bacterial genome assemblies from short and long sequencing reads. PLoS Comput Biol 13:e1005595. doi:10.1371/journal.pcbi.1005595 28594827 PMC5481147

[9] Kapatai G , Sheppard CL , Al-Shahib A , Litt DJ , Underwood AP , Harrison TG , Fry NK . 2016. Whole genome sequencing of Streptococcus pneumoniae: development, evaluation and verification of targets for serogroup and serotype prediction using an automated pipeline. PeerJ 4:e2477. doi:10.7717/peerj.2477 27672516 PMC5028725

[10] Zhou M , Wang Z , Zhang L , Kudinha T , An H , Qian C , Jiang B , Wang Y , Xu Y , Liu Z , Zhang H , Zhang J . 2021. Serotype distribution, antimicrobial susceptibility, multilocus sequencing type and virulence of invasive Streptococcus pneumoniae in China: a six-year multicenter study. Front Microbiol 12:798750. doi:10.3389/fmicb.2021.798750 35095809 PMC8793633

[11] Feil EJ , Li BC , Aanensen DM , Hanage WP , Spratt BG . 2004. eBURST: inferring patterns of evolutionary descent among clusters of related bacterial genotypes from multilocus sequence typing data. J Bacteriol 186:1518–1530. doi:10.1128/JB.186.5.1518-1530.2004 14973027 PMC344416

[12] Page AJ , Cummins CA , Hunt M , Wong VK , Reuter S , Holden MT , Fookes M , Falush D , Keane JA , Parkhill J . 2015. Roary: rapid large-scale prokaryote pan genome analysis. Bioinf 31:3691–3693. doi:10.1093/bioinformatics/btv421 PMC4817141 26198102

[13] Song JH . 2013. Advances in pneumococcal antibiotic resistance. Expert Rev Respir Med 7:491–498. doi:10.1586/17476348.2013.816572 24138693

[14] Liu B , Zheng D , Jin Q , Chen L , Yang J . 2019. VFDB 2019: a comparative pathogenomic platform with an interactive web interface. Nucleic Acids Res 47:D687–D692. doi:10.1093/nar/gky1080 30395255 PMC6324032

[15] Wang X , Cong Z , Huang W , Li C . 2020. Molecular characterization of Streptococcus pneumoniae isolated from pediatric patients in Shanghai, China. Pediatr Pulmonol 55:2135–2141. doi:10.1002/ppul.24877 32470194

[16] Yan Z , Cui Y , Huang X , Lei S , Zhou W , Tong W , Chen W , Shen M , Wu K , Jiang Y . 2021. Molecular characterization based on whole-genome sequencing of Streptococcus Pneumoniae in children living in Southwest China during 2017-2019. Front Cell Infect Microbiol 11:726740. doi:10.3389/fcimb.2021.726740 34796125 PMC8593041

[17] Weinstein MP . 2021. Performance standards for antimicrobial susceptibility testing M100. Clinical and laboratory standards institute, Wayne, PA 19087 USA.

[18] Curtin F , Schulz P . 1998. Multiple correlations and Bonferroni's correction. Biol Psychiatry 44:775–777. doi:10.1016/s0006-3223(98)00043-2 9798082

[19] Wahl B , O’Brien KL , Greenbaum A , Majumder A , Liu L , Chu Y , Lukšić I , Nair H , McAllister DA , Campbell H , Rudan I , Black R , Knoll MD . 2018. Burden of Streptococcus pneumoniae and Haemophilus influenzae type B disease in children in the era of conjugate vaccines: global, regional, and national estimates for 2000-15. Lancet Glob Health 6:e744–e757. doi:10.1016/S2214-109X(18)30247-X 29903376 PMC6005122

[20] Zhu X , Ge Y , Wu T , Zhao K , Chen Y , Wu B , Zhu F , Zhu B , Cui L . 2020. Co-infection with respiratory pathogens among COVID-2019 cases. Virus Res 285:198005. doi:10.1016/j.virusres.2020.198005 32408156 PMC7213959

[21] Huang LD , Yang MJ , Huang YY , Jiang KY , Yan J , Sun AH . 2022. Molecular characterization of predominant serotypes, drug resistance, and virulence genes of Streptococcus pneumoniae isolates from East China. Front Microbiol 13:892364. doi:10.3389/fmicb.2022.892364 35722327 PMC9198556

[22] Wang Q , Shi W , Li Y , Gao W , Yuan L , Dong F , Yao K . 2020. Serotype distribution of Streptococcus pneumoniae isolated from children hospitalized in Beijing children's hospital (2013-2019). Vaccine 38:7858–7864. doi:10.1016/j.vaccine.2020.10.005 33164807

[23] Walekhwa M , Muturi M , Gunturu R , Kenya E , Kabera B . 2018. Streptococcus pneumoniae serotype epidemiology among PCV-10 vaccinated and unvaccinated children at gertrude’s children’s hospital F1000Res 7:879. doi:10.12688/f1000research.14387.1 PMC6367659 30800286

[24] Goh SL , Kee BP , Abdul Jabar K , Chua KH , Nathan AM , Bruyne J , Ngoi ST , Teh CSJ . 2020. Molecular detection and genotypic characterisation of Streptococcus pneumoniae isolated from children in Malaysia. Pathog Glob Health 114:46–54. doi:10.1080/20477724.2020.1719325 32003298 PMC7144269

[25] Suaya JA , Mendes RE , Sings HL , Arguedas A , Reinert RR , Jodar L , Isturiz RE , Gessner BD . 2020. Streptococcus pneumoniae serotype distribution and antimicrobial nonsusceptibility trends among adults with pneumonia in the United States, 2009‒2017. J Infect 81:557–566. doi:10.1016/j.jinf.2020.07.035 32739491

[26] Pick H , Daniel P , Rodrigo C , Bewick T , Ashton D , Lawrence H , Baskaran V , Edwards-Pritchard RC , Sheppard C , Eletu SD , Rose S , Litt D , Fry NK , Ladhani S , Chand M , Trotter C , McKeever TM , Lim WS . 2020. Pneumococcal serotype trends, surveillance and risk factors in UK adult pneumonia, 2013-18. Thorax 75:38–49. doi:10.1136/thoraxjnl-2019-213725 31594801

[27] Yanagihara K , Kosai K , Mikamo H , Mukae H , Takesue Y , Abe M , Taniguchi K , Petigara T , Kaku M . 2021. Serotype distribution and antimicrobial susceptibility of Streptococcus pneumoniae associated with invasive pneumococcal disease among adults in Japan. Int J Infect Dis 102:260–268. doi:10.1016/j.ijid.2020.10.017 33065297

[28] Morales M , Ludwig G , Ercibengoa M , Esteva C , Sanchez-Encinales V , Alonso M , Muñoz-Almagro C , Marimón JM . 2018. Changes in the serotype distribution of Streptococcus pneumoniae causing otitis media after Pcv13 introduction in Spain. PLoS One 13:e0209048. doi:10.1371/journal.pone.0209048 30562385 PMC6298674

[29] Lane JR , Tata M , Briles DE , Orihuela CJ . 2022. A Jack of all trades: the role of pneumococcal surface protein A in the pathogenesis of Streptococcus pneumoniae. Front Cell Infect Microbiol 12:826264. doi:10.3389/fcimb.2022.826264 35186799 PMC8847780

[30] Jedrzejas MJ . 2001. Pneumococcal virulence factors: structure and function. Microbiol Mol Biol Rev 65:187–207. doi:10.1128/MMBR.65.2.187-207.2001 11381099 PMC99024

[31] Rivera AM , Boucher HW . 2011. Current concepts in antimicrobial therapy against select gram-positive organisms: methicillin-resistant Staphylococcus aureus, penicillin-resistant Pneumococci, and vancomycin-resistant Enterococci. Mayo Clin Proc 86:1230–1243. doi:10.4065/mcp.2011.0514 22134942 PMC3228624

[32] van de Beek D , Cabellos C , Dzupova O , Esposito S , Klein M , Kloek AT , Leib SL , Mourvillier B , Ostergaard C , Pagliano P , Pfister HW , Read RC , Sipahi OR , Brouwer MC , ESCMID Study Group for Infections of the Brain (ESGIB) . 2016. ESCMID guideline: diagnosis and treatment of acute bacterial meningitis. Clin Microbiol Infect 22 Suppl 3:S37–S62. doi:10.1016/j.cmi.2016.01.007 27062097

